# Estimation of radiological impact of the activities of Olusosun Dump Site on workers and dwellers of Olusosun, in Lagos Southwest Nigeria

**DOI:** 10.1093/jrr/rrac067

**Published:** 2022-11-14

**Authors:** Kayode Idowu Ogungbemi, Margret Bose Adedokun, Ayo Zaccheaus Ibitoye, Olusola Olurotimi Oyebola, Ronke Latifat Owoade

**Affiliations:** Department of Physics, University of Lagos Akoka-Yaba, Lagos 100213, Nigeria; Department of Physics, University of Lagos Akoka-Yaba, Lagos 100213, Nigeria; Department of Radiation Biology, Radiotherapy, Radiodiagnosis and Radiography, College of Medicine, Lagos University Teaching Hospital, Idi-Araba, Lagos 100254, Nigeria; Department of Physics, University of Lagos Akoka-Yaba, Lagos 100213, Nigeria; National Institute of Radiation Protection and Research University of Ibadan, Ibadan 200213, Nigeria

**Keywords:** Olusosun dump site, natural radioactivity, radiological hazard indices, soil, water

## Abstract

This study assessed the potential radiological risks associated with the activities on Olusosun dump site on workers and dwellers of Olusosun community. The activity concentrations of ^238^U, ^232^Th and ^40^K from of soil and water samples were determined using High-Purity Germanium (HPGe) detector. The background radiation level of Olusosun dump site was measured using a portable Geiger-Müller counter-Radeye B20 survey meter. The mean value of background radiation was 1.46 mSv/yr. This value is about 46% higher than the recommended reference level of 1.0 mSv/yr for the public. The mean activity concentrations of ^238^U, ^232^Th and ^40^K in the soil samples were 19.1 ± 3.2, 29.1 ± 4.4 and 171.5 ± 6.1 Bq/kg respectively which are about 45.4%, 35.3% and 59.2% lower than the world’s average levels. For the water samples, the mean activity concentrations obtained for ^238^U, ^232^Th and ^40^K are 0.4 ± 0.4, 0.8 ± 0.2 and 0.8 ± 0.3 Bq/l respectively. These are about 99.9% and 20% lower than WHO reference levels for ^238^U and ^232^Th. The mean absorbed dose rate in air (D), Annual effective dose (AED) outdoor, Radium Equivalent (R_eq_), External hazard index (H_ex_) internal hazard index (H_in_) and Excess lifetime cancer risk (ELCR) from soil samples were 33.6 nGy/h, 41.0 μSv/yr, 73.1 Bq/kg and 0.2, 0.3 and $1.4\times{10}^{-4}$, respectively. Absorbed dose in air D, AED outdoor, R_eq_, H_ex_, H_in_ and ELCR are 41.1%, 41.4%, 80.3%, 80%, 75% and 50% lower than their corresponding world’s average and references. The estimated AEDw from ingestion of water is 148.9 ± 50.4 μSv/yr, this is about 49% higher than the WHO reference level of 100.0 μSv/y from ingestion of water. The radiological hazard indices estimated from soil samples do not indicate any potential risks to the users. The elevated background radiation level of the dump site, and AEDw from ingestion of water, however, suggest that the activities on Olusosun dump site pose potential radiological risks on workers on the site and the public from consumption of water from Olusosun community.

## INTRODUCTION

Human activities generate waste, and poor waste management and disposal are of great concern as they pose several challenges to the well-being of city residents. Industrialization and population growth in most cities of the world result in changes in the quantity, composition and quality of waste generated, this can be observed in most cities of developing countries [1], such as Nigeria. Waste generated in the vicinity of industries are of ecological importance because the waste can be a source of potential pollution to water, food sources, land, air and vegetation [2].

In the mega city of Lagos, Olusosun is an area with a dump site of about 100-acres. Lagos is the most economically significant state in Nigeria and has a high-industrialized status [3]. The Olusosun dump site is the largest in Africa and one of the largest in the world [4]. Olusosun has both residential and industrial buildings, waste from about 500 containers ships are delivered to this site generating about 10 000 tons of solid waste per day. [5]. Waste is generated from diverse sources, ranging from industrial to domestic waste, electronic waste, waste from hospitals, etc. The size of the Olusosun dump site and the industrial activities in Lagos, mean that the dump site has the potential to be rich with toxic materials. Toxics metals and naturally occurring radionuclide from dump sites can be transported to and accumulated in environmental media (such as air, water sources and soil in and around waste dump sites) in significant amounts. When waste with elevated levels of radionuclides finds its way to a dump site, this can result in environmental pollutions: leading to contamination of air, sources of water supply and farmlands (through leaching and surface runoff) around the dump sites. Enhanced radiation in these environmental media will lead to elevated background radiation, elevated external and internal exposure to the population.

**Fig. 1:**
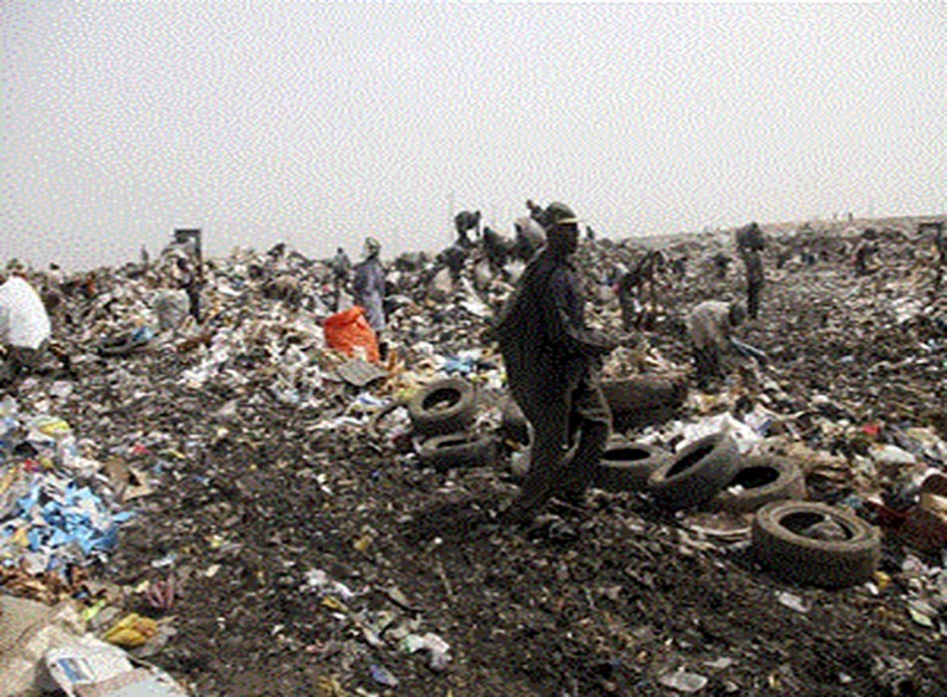
Busy Olusosun Dump site.

Olusosun dump site has been active since 1989 [6]. Waste of all kinds from all over Lagos is deposited at this site. The site is always busy with constant release of toxic fumes from recycling activities. There is a concern about elevated radiation levels within the Olusosun community due to the activities at the dump site. Elevated radiation levels in this area will affect the health of workers on the site and members of the Olusosun community. Many people come scavenging for scrap materials that can be picked and sold for recycling purposes (Fig. 1). These people focus on possible income without the knowledge of the possible high levels of radiation they could be exposed to.

External radiation exposure pathways to the population have been extensively studied and radioactivity measurements in areas around dump sites have shown the existence of traces of radionuclides in the staple foods consumed in Nigeria [7, 8] and because of this, refuse dump sites were identified as liable recipients in contaminants of radioactive materials. Studies on the levels of radiation contamination in soil, water and vegetation due to industrial activities in Lagos have also been conducted, and the study revealed potential radiological risks to the health of the public in the Lagos metropolis [9].

Over 80% of the public exposure to radiation comes from natural radionuclides such as ^238^U, ^232^Th and ^40^K, their progenies and cosmic radiation [10]. Some of the exposure pathways of radionuclides to the population are ingestion of contaminated food and water and through inhalation. The activities on the Olusosun dump site have the potential to accumulate radionuclides in the soil, elevate radioactivity in the air and water bodies (through leaching and water runoff) of the Olusosun metropolis. To adequately quantify and determine the risk to the population in this area, it is imperative to carry out measurements of different environmental media in Olusosun community. Assessments of the radioactivity levels of the soil from the Olusosun dump site has been carried out [11], but an accurate measurement of background radiation and radionuclide levels in soil and water samples from within and in the vicinity of Olusosun will provide information from which the current average radiation exposure to the public from this dump site can be estimated. The aim of this study therefore is to assess the radiological effects associated with activities on the Olusosun dump site to those working on the site, those living in the vicinity and the public by measuring the activity concentrations of ^40^K, ^228^Th and ^238^U from soil and water samples collected from various locations in and around the dump site.

## MATERIALS AND METHODS

### Study location

**Fig. 2:**
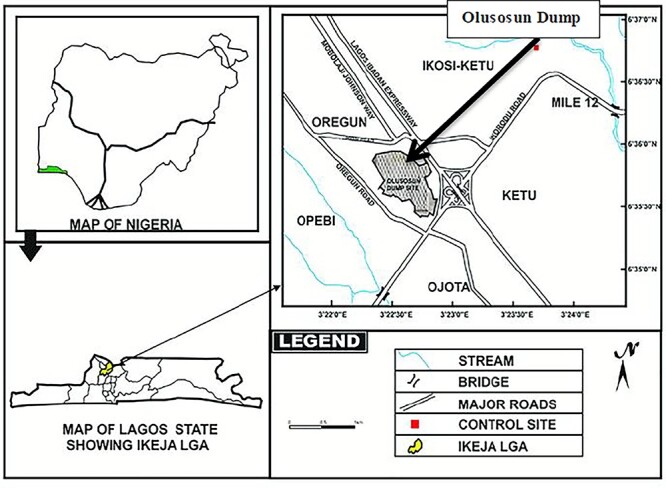
Map of the study site (adapted from [15]).

Olusosun landfill (Fig. 2) is in Lagos, South-Western, Nigeria. The landfill lies within the latitude 6°35′N and longitude 3°45′E. About 15 000 residential homes exist near and around the site [4], occupied by substantial populations who work on the site and in the industries around the area.

### Sample collection and preparation

Water and soil samples were collected from 15 strategic locations (Table 1) in the vicinity of the Olusosun dump site community.

**Table 1:** Water and soil sampling locations around the study area

Sample Locations	Code	Sample Locations	Code
Olusosun Mosque	OLM	Olusosun Recycling Center	ORC
Center of Dump Site	COD	Resident close to Total Oil	RTO
Nylon Dump	NDP	Apostolic Faith Church	AFC
Dump Entrance	DEE	Dump Site Exit	DSE1
LAWMA Office	LAO	Ule Baba Egba	UBE
Olusosun Church	OLC	Dump Site Exit 2	DSE2
Can Dumping Point	CDP	Dump Site Exit 3	DSE3
Huge Metal point	HMP		

### Soil samples

A total of 15 soil samples were collected at strategic locations around the dump site and at close vicinity to the Olusosun metropolis (Table 1). Each soil sample was collected at a depth 0–15 cm. The samples were packed in black plastic bags, labeled and then taken to the laboratory. Each sample was air dried for about four weeks in plastic trays until constant weight was attained. The samples were then pulverized using a laboratory pestle and mortar and sieved using a 2 mm mesh. The fine grain obtained after sieving were packed in plastic containers each weighing an average of about 200 g. The weighed samples were hermetically sealed and left in this state for four weeks to allow for secular equilibrium between ^238^U and ^232^Th and their corresponding progenies.

### Water samples

Fifteen water samples were also collected from the main sources of water supply (boreholes and wells) in the Olusosun metropolis. The samples were collected in pre-washed 750 ml polyethylene sampling bottles, each bottle was filled to the brim, labelled and transported to the laboratory. The water samples were acidified by adding 0.5 ml of concentrated HNO_3_ per liter water to prevent adsorption or loss of radium (Ra) isotopes around the walls of the sampling container as in [3]. In the laboratory, each sample was emptied into and filled a 500 ml standard Marinelli beaker, hermetically sealed and then stored for four weeks to allow for secular equilibrium between ^238^U and ^232^Th and their progenies before radionuclides analysis.

### Background radiation measurement

In this work, the *in situ* background radiation measurement approach was adopted to determine the background radiation of the study area. *In situ* background measurement of terrestrial gamma radiation assumes of its existence in a laterally uniform distribution in the environment. A portable survey meter Geiger-Müller counter (Radeye B20, Thermo Fisher Scientific, USA). It is a multipurpose surface contamination detector for alpha, beta and gamma radiation with configurable dose rate measurements with automatic switch used to determine the background radiation. The measurement was carried out in the midday hours to ascertain the maximum response of the survey meter at 1 m above the ground called the gonadal level of measuring ionizing radiation. The absorbed dose rate in air was measured 1 m above the surface at five different locations around the dump site. Measurement was made from the top of the dump hill at an intervals of 5 m to the bottom of the dump hill taking three different values at each point and the average values for each point were recorded**.**

### Gamma spectrometry system

The activity concentrations of ^40^K, ^238^U and ^232^Th in the soil and water samples were measured through gamma spectrometry using a High Purity Germanium (HPGe) detector. The HPGe detector used is of serial number 9744, model GC8023 has relative efficiency 80% and resolution 2.3 keV full width at half maximum (FWHM) at 1.33 MeV. The detector is constantly cooled with liquid nitrogen at -196°C to reduce leakage current to acceptable levels. The detector is coupled to a Canberra series 10 plus multichannel analyzer (MCA). It is housed in a 5 cm thick lead shield that minimizes the effect of natural background radiation. The detector is interfaced with the MCA that consists of an analogue to digital converter (ADC), internal amplifier and in-built high voltage power supply (HVPS). The efficiency of the detector is the proportionality relationship that relates the activity of the source being counted and the number of counts observed. The efficiency of detector is calibrated regularly using a standard reference source sample supplied by IAEA (No. MGS6M315) and efficiency curve generated using GENIE 2000 software. Efficiency is such that the centroids of photo peaks energies are matched with the corresponding channel numbers continuously**.** The absolute photo-peak efficiency e of the detector for ^40^K, ^228^Th and ^238^U gamma-ray spectrum lines were derived from the relationship between the gamma photo peak efficiency of the detector and the photopeak energy E.

The minimum detectable activity (MDA) in water and soil samples for ^40^K, ^238^U and ^232^Th of this detector are given in Table 2.

**Table 2:** MDA of detector

Radionuclide	Water (Bq/l)	Soil (Bq/kg)
^238^U	0.022	0.022
^232^Th	0.020	0.020
^40^K	0.080	0.080

### Activity measurement

For water and soil samples, gamma spectra were accumulated for a counting time of 28 800 s for each sample and the activity concentrations of ^40^K, ^238^U and ^232^Th in water and soil samples were obtained from the count rates from photo-peaks of interest. For ^238^U, the photo-peaks considered were photo-peaks of ^214^Pb and ^214^Bi of energies 295.21 keV and 609.31 keV respectively. For ^232^Th, the photo-peaks considered were the photo-peaks of ^212^Pb, ^228^Ac and ^208^Tl of energies 238.63 keV, 911.21 keV and 2614.55 keV. The activity concentration of ^40^K was determined from its photo-peak of energy1460.8 keV. Activity concentration in water and soil samples were determined using equations (1) and (2) respectively:

(1) \begin{equation*} A\left( Bq/l\right)=\frac{NC}{t^{\ast }{v}^{\ast }{\varepsilon}^{\ast }{P}_{\gamma }} \end{equation*}

(2) \begin{equation*} A\left( Bq/ kg\right)=\frac{NC}{t^{\ast }{m}^{\ast }{\varepsilon}^{\ast }{P}_{\gamma }} \end{equation*}

where A is the activity concentration of the radionuclide, NC is the net count for each radionuclide which is the gross count minus the background, t is the counting lifetime in seconds, v is the volume of water in liter, m is mass in kg, $\varepsilon$ is the detector energy dependent efficiency for each radionuclide, and ${P}_Y$ is the gamma-ray yield per disintegration of the nuclide (emission probability).

### Annual *effective dose due to ingestion of water*

To assess the radiation exposure to the public from drinking water in Olusosun community, the annual effective dose (AED) due to ingestion of U, Th and K in water samples was calculated using the activity concentration and dose conversion factors of ^238^U, ^232^Th and ^40^K using the expression of equation (3):

(3) \begin{align*}AE{D}_w=\sum \limits_{i=1}^3{A}_i DC{F}_i\textrm{I}\end{align*}

Where $AE{D}_w$ is the AED for water, ${A}_i$ is the activity concentration of radionuclide i, $DC{F}_i$ is the dose conversion factor for radionuclide i and I is the WHO annual water consumption for an adult = 730 l. DCf for ^238^U, ^232^Th and ^40^K are 4 x 10^−8^ Sv/Bq, ^232^Th is 2.3 x 10^−7^ Sv/Bq and 6.2 x 10^−9^ Sv/Bq, respectively [11–13].

### Absorbed dose rate in air

The absorbed dose rates (D) due to gamma radiation in air at 1 m above the ground have been obtained with use of the activity concentration using equation (3) [14].

(3) \begin{equation*} D\kern0.33em \left( nGy{h}^{-1}\right)\kern0.45em =0.462 AU+\kern0.33em 0.621 ATh+0.0417 AK \end{equation*}

Where D is the absorbed rate A_U_, A_Th_ and A_K_ are the activity concentrations of the radionuclide ^238^U, ^232^Th and ^40^K respectively.

### Annual effective dose rate (outdoor)

In evaluating the AED from soil samples, the absorbed dose in air, outdoor occupancy factor of 0.2 and the conversion factor of 0.7 SvGy were used as in equation (4) [15].

(4) \begin{equation*} AED=D\left( nGy{h}^{-1}\right)\times 8760\times 0.2\times 0.7 SvG{y}^{-1}\times{10}^{-6} \end{equation*}

Where AED is measured in mSy/yr, D is the absorbed dose rate in nGy/h from background gamma radiation, 8766 h in a year. Only the outdoor absorbed dose (absorbed dose in air) has been considered here. This is to evaluate the AED of workers during activities around the dump site.

### Radium equivalent activity

Radium equivalent activity (Ra_eq_) is a radiation hazard index used to assess the gamma radiation hazards associated with naturally occurring radionuclides materials that is ^238^U, ^232^Th and ^40^K. The distribution of ^40^K, ^238^U and ^232^Th in soil is not uniform to define their uniformity with respect to radiation exposures the radium equivalent activity (Ra_eq_) was introduced [14] and it was calculated using equation (5).

(5) \begin{align*} \text{Ra}_{\text{eq}} (\text{Bq/kg})\ = \ {\text{A}}_{\text{U}} + ({\text{A}}_{\text{K}} \times{\text{0.077}}) + ({\text{A}}_{\text{Th}} \times 1.43)\end{align*}

Where A_U_, A_Th_ and A_K_ are activity concentrations of ^238^U, ^232^Th and ^40^K respectively in Bq/kg.

### External hazard index

The external hazard index or outdoor radiation hazard index is denoted by H_ex_. H_ex_ is commonly used to evaluate radiation dose rate due to external exposure to gamma radiation from natural radionuclides in soil samples calculated using equation (6) [16]:

(6) \begin{equation*} {\text{H}}_{ex}=\frac{{\text{A}}_{\text{U}}}{370}+\frac{{\text{A}}_{\text{Th}}}{259}+\frac{{\text{A}}_{\text{K}}}{4810} \end{equation*}

Where A_u_, A_Th_ and A_K_ are the activity concentrations (Bqkg^−1^) of ^238^U, ^232^Th and ^40^K respectively. The value of this index should be less than 1.0, for the radiation hazard to be considered acceptable to the public.

### Excess lifetime cancer risk

Excess lifetime Cancer Risk (ELCR) is another radiological hazard index used in estimating the potential carcinogenic effects of exposure to radionuclides through inhalation of the dust and water consumption by workers and those living in the vicinity of Olusosun dump site. The ELCR was obtained from the AED outdoor according to [17], using equation (7):

(7) \begin{equation*} ELCR= AED\left(\frac{mSv}{yr}\right)\ast 70{yr}^{\ast }0.05\left(S{v}^{-1}\right) \end{equation*}

AED is in mSv/yr, life expectancy of 70 years has been used and fatal risk factor of 0.05 per Sievert (Sv^−1^).

## RESULTS AND DISSCUSSION

### *In situ* background measurement

Table 3 shows the results of the background radiation measurement (mSv/yr) made from the bottom(ground) of the dump site to 20.0 m away from the bottom at an interval of 5.0 m.

**Table 3:** Background radiation distribution at different distance from the ground at the dump site

Height(m)	Background radiation (mSv/yr)
0.0	0.8
5.0	0.8
10.0	1.3
15.0	2.0
20.0	2.6

As shown in Table 3, the study revealed that the background ionizing radiation levels of the study area ranged from 0.8 mSv/yr (at the ground (0 m) and at distance 5 m above the ground) to 2.6 mSv/yr (at distance 20 m above the ground). From 5 m above the ground, the background radiations increased with increase in the distance from the ground, which could be due to cosmic ray influence. The overall mean background radiation measured at the dump site is 1.5 mSv/yr. This obtained mean value is about 97% below the ICRP recommended limiting occupational radiation exposure of 50.0 mSv/yr but 46% higher than the 1.0 mSv/yr [18] recommended reference level for members of the public.

Tables 4 presents the activity concentrations of the ^238^U, ^232^Th and ^40^K from soil samples from all researched locations. ^238^U and ^232^Th were detected in all the soil samples. Activity concentration of ^238^U in the soil samples ranged between 11.1 Bq/kg (DSE3) and 36.8 Bq/kg (DSE2). Activity concentrations of ^232^Th ranged from 3.9 Bq/kg (DSE3) and 60.1 Bq/kg (OLM). ^40^K was detected in all the samples except in the sample from COD. Activity concentration of ^40^K ranged from below detectable level (BDL) to 840.3 Bq/kg. The highest activity concentration of 840.3 Bq/kg was recorded from the sample from AFC. The mean activity concentrations of ^238^U, ^232^Th and ^40^K in the soil samples are 19.1 ± 3.2, 29.1 ± 4.4 and 171.5 ± 6.1 Bq/kg respectively which are about 54.6%, 64.7% and 40.8% of their respective world’s average levels according to [19]

**Table 4:** Activity concentrations of natural radionuclides in soil samples

Sample Code	^238^U (Bq/kg)	^232^Th(Bq/kg)	^40^K (Bq/kg)
OLM	23.3 ± 3.2	60.1 ± 5.1	33.2 ± 2.6
COD	25.3 ± 1.8	55.6 ± 5.9	BDL
NDP	14.5 ± 1.1	22.5 ± 2.6	154.2 ± 4.9
DEE	12.8 ± 1.8	25.9 ± 4.1	72.7 ± 3.6
LAO	26.2 ± 3.0	56.2 ± 6.9	120.00 ± 8.7
OLC	13.8 ± 1.6	22.4 ± 3.2	187.8 ± 5.9
CDP	14.1 ± 2.3	19.4 ± 2.6	91.5 ± 5.3
HMP	22.2 ± 1.8	31.2 ± 2.2	119.1 ± 5.2
ORC	17.2 ± 1.9	32.6 ± 4.2	134.6 ± 6.8
RTO	24.4 ± 2.1	35.8 ± 4.1	219.3 ± 6.8
AFC	14.3 ± 0.2	11.9 ± 0.0	840.3 ± 10.3
DSE1	17.1 ± 8.8	17.1 ± 7.5	179.1 ± 8.1
UBE	13.5 ± 6.4	9.9 ± 6.8	83.0 ± 7.0
DSE2	36.8 ± 6.4	32.2 ± 3.9	19.7 ± 9.5
DSE3	11.1 ± 5.1	3.9 ± 7.0	146.0 ± 0.5
**Mean**	19.1 ± 3.2	29.1 ± 4.4	171.5 ± 6.1
**world’s average level**	35.0	45.0	420.0

#### NA- not applicable, this is because there is no reference level for ^40^K

The activity concentrations of ^238^U, ^232^Th and ^40^K in water samples are shown in Table 5. In the water samples, ^232^Th was detected in all. Activity concentration of ^238^U in the water sample ranged between BDL and 1.0 Bq/l, ^232^Th ranged between 0.5 and 1.4 Bq/l and ^40^K ranged between BDL and 1.7 Bq/l. In water, the mean activity concentration of ^232^Th was the highest followed by the mean activity concentration of ^40^K in the least was the mean activity concentration of ^238^U. The mean activity concentration obtained for ^238^U, ^232^Th and ^40^K are 0.4 ± 0.4, 0.8 ± 0.2 and 0.8 ± 0.3 Bq/l respectively. The values obtained for ^238^U and ^232^Th are about 99.9 and 20% lower than their WHO reference levels of 10.0 and 1.0 Bq/l respectively [20].

**Table 5:** Activity concentrations (Bq/l) of natural radionuclides in water

Sample Code	^238^U(Bq/l)	^232^Th (Bq/l)	^40^K(Bq/l)
OLM	BDL	0.6 ± 0.1	BDL
COD	1.0 ± 0.9	1.4 ± 0.1	0.3 ± 0.1
NDP	BDL	0.5 ± 0.1	BDL
DEE	BDL	0.7 ± 0.2	BDL
LAO	BDL	0.8 ± 0.2	BDL
OLC	0.5 ± 0.5	0.9 ± 0.1	1.4 ± 0.4
CDP	0.1 ± 0.3	0.6 ± 0.3	0.4 ± 0.1
HMP	0.2 ± 0.9	0.5 ± 0.1	BDL
ORC	1.0 ± 0.9	1.4 ± 0.8	0.3 ± 0.1
RTO	0.1 ± 0.1	1.0 ± 0.0	0.7 ± 0.3
AFC	0.2 ± 0.1	0.7 ± 0.4	0.7 ± 0.9
DSE1	0.1 ± 0.1	0.9 ± 0.2	0.5 ± 0.2
UBE	0.5 ± 0.5	0.91 ± 0.65	1.4 ± 1.0
DSE2	0.2 ± 0.1	0.7 ± 0.1	0.4 ± 0.1
DSE3	0.2 ± 0.1	0.8 ± 0.1	1.7 ± 0.0
**Mean**	**0.4 ± 0.4**	**0.8 ± 0.2**	**0.8 ± 0.3**
**Guidance level**	**10.0**	**1.0**	**NA**

BLD – below the detectable limits

NA – not applicable

Comparison of the obtained of activity concentration of ^238^U, ^232^Th and ^40^K in soil samples with values from literature are as presented on Table 6. The results of [21–25] are within the range obtained for the activity concentration ^238^U, while the obtained mean activity concentration for ^238^U for the current study is only about 34%, 43%, 35%, 38% and 30% of the results of [11]; (24(Amman)); [26–28] for ^238^U respectively. The range of activity concentration obtained for ^232^Th in soil in this study is comparable to the results from all compared similar studies except for the results of [25, 28]. For which obtained value is lower. Since studies carried out in Ibadan [25, 28], the high values could be attributed to the geology of Ibadan. The mean value obtained from this study is about 2.4 and 2 times higher than the values by [11] which is a study carried earlier in the same location as the present study. This result shows that ^232^Th has accumulated in Olusosun over time. The range of activity concentration obtained for ^40^K in soil in this study is comparable to the results from all compared similar studies. The mean activity concentration obtained from this study is however less than 50% of the result obtained by [11] in the same location as this study. This therefore implies that activity concentrations of ^238^U and ^40^K have depreciated in the soil of Olusosun while the activity concentration of ^232^Th has appreciated.

**Table 6:** Comparison of the obtained value of activity concentration of radionuclides in soil samples with values from similar studies

Study location	Bq/kg			Reference
	^238^U	^232^Th	^40^K	
Taiwan	30.0	44.0	431.0	[21]
Instanbul,Turkey	21.0	37.0	342.0	[22]
Rajasthan, India	30–78.0	43–106.0	50–137.0	[23]
Amman, Jordan	56.4	28.8	501.0	[24]
Karak, Jordan	22.9	27.2	410.0	[24]
Taiwan	54.0	32.4	794.0	[25]
Agaba-Amman	44.4	36.3	208.0	[26]
University of Ibadan	50.0	261.4	261.0	[27]
Olusosun	65.5	13.3	408.0	[11]
Polytechnic Ibadan	13.0	126.0	125.0	[28]
Olusosun	19.1	29.1	171.5	**Present study**

Table 7 presents the estimated AED from ingestion of water from Olusosun community. AEDw ranged from 89.0 ± 23.5 μSv from NDP to 975.7 ± 40.3 μSv from HMP. The AEDw from all the water samples except the one collected from NDP were higher than the WHO’s reference level of 100.0 μSv [29]. The average value from all samples was 148.9 ± 50.4 μSv which is about 49% higher than the WHO’s reference level of AED from ingestion of water. These results suggest that the activities on the Olusosun dump site have radiological effects on the water used by the Olusosun community. In terms of radiation protection, there are potential radiological effects to the public associated with drinking water from Olusosun community.

**Table 7:** AED from sampled water

Sample Code	AEDw(μSv)
OLM	102.4 ± 23.5
COD	265.6 ± 47.6
NDP	89.0 ± 23.5
DEE	110.8 ± 30.2
LAO	134.3 ± 25.2
OLC	176.0 ± 40.8
CDP	106.6 ± 54.3
HMP	975.7 ± 40.3
ORC	265.6 ± 167.6
RTO	169.8 ± 11.2
AFC	119.8 ± 80.0
DSE1	155.9 ± 32.6
UBE	176.0 ± 131.3
DSE2	121.3 ± 21.4
DSE3	142.3 ± 26.6
**Mean**	148.9 ± 50.4
**Reference level**	100.0

The AED from the ingestion of water from the Olusosun community has been compared with results of other studies on radioactivity levels of drinking water from different countries; this is presented in Table 8. The level AED from ingestion of water has been shown to vary by countries. Different studies in the same countries; Nigeria [30, 31], Ghana [12, 32] and Iraq [33–35] also had varying results. The result from the current study is higher than the results reported from Ghana (13:32), Nigeria [30], Egypt [36], Saudi Arabia [37], Malaysia [38] and Iraq [35] but lower than the results from Brazil [39], Nigeria [31] and Iraq [33, 34]. Although the AED from the ingestion of water (AED_w_) from this study is above the guidance level of WHO, it is within the range of values reported in literature.

**Table 8:** Comparison of the mean AED from ingestion of water from Olusosun community with results from similar studies

Country	AED μSv/yr	Reference
Brazil	800.0	[39]
Nigeria	120.0	[30]
Ghana	113.0	[12]
	76.6	
Ghana	1.8	[32]
Nigeria	10250.0	[31]
Egypt	58.0	[36]
Saudi Arabia	58.0	[37]
Iraq	269.0	[33]
Malaysia	9.6	[38]
Iraq	252.0	[34]
Iraq	13.0	[35]
Nigeria	148.9	present study

The obtained activity concentrations of ^238^U, ^232^Th and ^40^K were used to evaluate the radiological parameters from the samples Tables 9. The radioactive contamination of air and soil can be transferred to humans through inhalation and food via plants’ uptake while the radioactive contamination from water can be transferred to humans through ingestion. The radiological parameters accessed for each sample type have been chosen according to the channels for which the population is exposed to NORM from water and from soil. AED has been considered for water samples while D, AED, ELCR, R_eq_, H_ex_ and H_in_ have been considered for soil. Absorbed dose D gives a direct connection between terrestrial gamma radiation and radionuclide concentrations and AED is used to assess the potential for radiochemical changes in specific tissues thus their usefulness. R_eq_ and H_ex_ are parameters used in evaluating radioactivity from soil, R_eq_ generalizes the exposure to ^238^U, ^232^Th and ^40^K, H_ex_ is used to evaluate the external gamma radiation dose and ELCR helps to estimate the potential carcinogenic effects of exposure to radionuclides through inhalation.

**Table 9:** Radiological hazard parameters from soil samples

Sample Code	Absorbed Dose Rate (nGy/h)	Annual Effective Dose outdoor (μSv/yr)	Radium Equivalent Activity (Bq/kg)	External Hazard Index	Internal Hazard Index	Excess lifetime Cancer Risk x10^−4^
OLM	49.4	61.0	111.8	0.3	0.4	2.1
COD	46.1	57.0	104.8	0.3	0.4	2.0
NDP	27.1	33.0	58.6	0.2	0.2	1.2
DEE	25.0	31.0	55.5	0.2	0.2	1.1
LAO	52.0	64.0	115.8	0.3	0.4	2.2
OLC	28.1	34.0	60.3	0.2	0.2	1.2
CDP	22.4	27.0	49.0	0.1	0.2	0.9
HMP	34.6	42.0	76.0	0.2	0.3	1.5
ORC	33.8	41.0	74.2	0.2	0.3	1.4
RTO	42.6	52.0	92.4	0.3	0.3	1.8
AFC	49.0	60.0	96.0	0.3	0.3	2.1
DSE1	26.0	32.0	55.4	0.2	0.2	1.1
UBE	15.8	19.0	34.0	0.1	0.1	0.7
DSE2	37.8	46.0	84.3	0.2	0.3	1.6
DSE3	13.6	17.0	27.9	0.1	0.1	0.6
Mean	33.6	41.0	73.1	0.2	0.3	1.4
world’s average level	57.0	70.0	NA	NA	NA	NA
reference level	NA	NA	370.0	1.0	1.0	2.9

**Table 10:** Comparison of average radiological parameters from the soil of Olusosun with results of similar studies

Country	Ra_eq_ (Bq/kg)	D (nGy/h)	AED (μSv/y	Hex	Hin	Reference
Nigeria	73.1	33.6	41.0	0.2	0.3	present study
Nigeria	121.3	56.4	276.7	−	−	[11]
Palestine	43.0	20.5	25.0	0.1	0.2	[40]
Malaysia	214.3	87.5	106.0	0.5	−	[41]
China	202.6	178.8	880.0	0.6	0.6	[42]
Pakistan	136.1	64.6	120.0	0.4	0.5	[43]
Turkey	112.0	104.0	510.0	0.3	0.4	[44]
India	300.0	138.0	170.0	0.8	1.0	[45]
Taiwan	98.2	45.6	57.2	0.3	0.3	[46]

From Table 9, for the soil samples, absorbed dose rate in air ranged between 13.6 and 52.0 nGy/h, AED (outdoor) obtained ranged from 17.0 to 64.0 μSv/yr with average values of 33.6 nGy/h and 41.0 μSv/yr, respectively. The mean value for D is about 41.1% lower than its world’s average level of 57.0 nGy/h [16]. Although the mean AED is about 41.4% lower the average world’s level of 70.0 μSv/yr [16] for AED outdoor, AED from OLM, COD, LAO, RTO, AFC and DSE2 were higher than the overall average of 41.0 μSvyr^−1^. These locations have higher potential to radiation effects in the area than the other points considered. Higher ECLR and H_ex_ values were obtained from points OLM, COD, LAO, RTO, AFC and DSE2.

The radium equivalent activity obtained from the soil ranged from 27.9 to 115.8, external hazard index ranged from 0.1 to 0.3, internal hazard index ranged from 0.1 to 0.4 with mean values of 73.1 Bq/kg, 0.2 and 0.3, respectively. The mean R_eq_ and H_ex_ are each about 80% lower than the world’s average level of 370.0 Bq/kg [16] and the reference level of 1.0 [16] respectively, while H_in_ is 75% lower than its reference level of 1.0 [16]. The values of ELCR from the soil samples ranged from 0.6 to $2.2\times{10}^{-4}$ with an average value of $1.4\times{10}^{-4}$. The mean value of ELCR is about 50% lower than the world’s average levels of $2.9\times{10}^{-4}$ for ELCR from all carcinogens [9]. All assessed radiological hazard parameters from the soil samples are lower than their respective reference levels or world’s average levels. The soil from the Olusosun dump site and its environment do not indicate significant radiological concern and their use does not pose any potential radiological hazard to the public.

Table 10 shows a comparison of the radiological parameters obtained in this study and other similar from different other countries. The mean values of Ra_eq,_ D and AED in the current study were all lower than those reported from an earlier study in the same location [11]. The lower value of Ra_eq_ suggests that natural radionuclides have not accumulated in the soil of Olusosun, this could be because of transportation process of the radionuclides. The lower values of D and AED are not out of place as these parameters are exposure in air and air is not stagnant; status of a location may not depend on the status of the location in the past. The average values of Ra_eq_, D, AED, H_ex_ and H_in_ in this study are higher than the values reported for Palestine [40] but lower than the values reported for Malaysia [41], China [42], Pakistan [43], Turkey [44], India [45] and Taiwan [46]. The radiological parameters in this study are therefore within the range of values reported in literature from similar studies and they are therefore not of significant radiological concern.

## CONCLUSION

The measurements of the background radiation, the activity concentrations of ^238^U, ^232^Th and ^40^K in soil and water from Olusosun dump site and community have been carried out. This was to assess the radiological health implications of the activities on the Olusosun dump site on workers of the site and dwellers of Olusosun community. The mean background radiation of the Olusosun dump site has been found to be lower than the 50.0 mSv/yr for the occupational dose reference level but higher than the 1.0 mSv/yr reference level for the public. The mean activity concentrations of ^238^U, ^232^Th and ^40^K from the soil from Olusosun dump site and its environs are within the ranges of results from other similar studies and less than the world’s average levels of 35.0, 45.0, 420.0 Bq/kg. Comparing results from this study with a previous study [11] at Olusosun community revealed that activity concentrations of ^238^U and ^40^K have depreciated in the soil of Olusosun while the activity concentration of ^232^Th has appreciated over time. The mean activity concentrations of ^238^U and ^232^Th in sampled water are both less than their 1 Bq/l reference level. All assessed radiological hazard parameters from the soil samples are lower than their respective reference levels/world’s average levels. The use of the soil from Olusosun does not pose significant radiological risks to workers and members of the Olusosun community. However, the activities of the Olusosun dump site have potential radiological risks to the population as the background radiation at the dump site and the AED from ingestion of water from Olusosun community are higher than their respective reference levels for the public.

## Data Availability

The data that supports the findings of this study are available within the article.

## References

[1] Ferronato N, Torretta V. Waste mismanagement in developing countries: A review of global issues. Int J Environ Res Public Health 2019;16:1060–88. 30909625 10.3390/ijerph16061060 PMC6466021

[2] Ogungbemi KI, Oyebola OO. Investigation of lead concentration in cow teeth within Otta an Urban Town of Abeokuta. Ilorin J Sci 2016;3:168–76.

[3] Adedokun MB, Aweda MA, Ogungbemi KI et al. Assessment of naturally occurring radionuclides in irrigation water from selected vegetable farms in Lagos, Nigeria. IFE J Sci 2018;20:607–15.

[4] Jenkins C. , 2011. An Incredible Satellite Tour of 15 Trash Dumps that are Bigger than Towns, http://www.businessinsider.com.au (20 December 2021, date last accessed).

[5] Freeman, A. , 2012. Seven of the Largest Landfills in the World. Takepart.com https://en.wikipedia.org/wiki/Olusosun_landfill (2 January 2022, date last accessed).

[6] Oyeku OT, Eludoyin AO. Heavy metal contamination of groundwater resources in a Nigerian urban settlement. Afr J Environ Sci Technol 2010;4:201–14.

[7] Akinloye MK, Olomo JB. The radioactivity in some grasses in the environment of nuclear research facilities located within the OAU, Ile-Ife, Nigeria. Niger J Phys 2005;17:219–25.

[8] Jibiri NN, Farai IP, Alausa SK. Activity concentration of ^226^Ra, ^228^Th and ^40^K in different food crops from a high background radiation area in Bisichi Jos Plateau State, Nigeria. Radiat Environ Biophys 2007;46:53–9. 17211656 10.1007/s00411-006-0085-9

[9] Adedokun MB, Aweda MA, Maleka PP et al. Natural radioactivity contents in commonly consumed leafy vegetables cultivated through surface water irrigation in Lagos state, Nigeria. J Radiat Res Appl Sci 2019;12:147–56.

[10] Farai IP, Jibiri NN. Baseline studies of terrestrial outdoor gamma dose rate levels in Nigeria. Radiat Prot Dosim 2000;88:247–54.

[11] Oladapo OO, Oni EA, Olawoyin AA et al. Assessment of natural radionuclides level in wasteland soils around Olusosun dumpsite Lagos, Nigeria. J Appl Phys 2012;2:38–43.

[12] Nguelem EJM, Darko EO, Ndontchueng MM et al. Assessment of natural radioactivity level in groundwater from selected areas in Accra Metropolis. Res J Environ Earth Sci 2013;5:85–93.

[13] World Health Organization (WHO) . Guidelines for Drinking-Water Quality, 4th edn. Geneva, Switzerland, 2011, 211–21 ISBN 978 92 4 1548151.

[14] United Nations Scientific Committee on the Effects of Atomic Radiation (UNSCEAR) . Exposures from natural radiation sources. In: Scientific Annex B. United Nations, Vol. I. New York: UNSCEAR, 2000.

[15] Oziegbe O, Oluduro AO, Oziegbe C et al. Assessment of heavy metal bioremediation potential of bacterial isolates from landfill. Saudi J Biol Sci 2021;28:3948–56. 34220251 10.1016/j.sjbs.2021.03.072 PMC8241888

[16] United Nations Scientific Committee on the Effects of Atomic Radiation UNSCEAR . *Report of the* General Assembly, with Scientific Annexes *2000*. Sources and effects of ionizing radiation volume I https://www.unscear.org/docs/reports/2010/UNSCEAR_2010_Report_M.pdf (28 July 2021, date last accessed).

[17] Hamzah Z, Saat A, Mashuri NH et al. Surface radiation dose and radionuclide measurement in ex-tin mining area, Kg Gajah, Perak. Malaysian J Anal Sci 2008;12:419–31.

[18] ICRP Publication . Annals of the ICRP Compendium of Dose Coefficients based on ICRP 60. 2001. 10.1016/j.icrp.2012.06.038 23025851

[19] World Nuclear Association . What Is Radiation. World Nuclear Association 2015. https://www.world-nuclear.org/ (24 February 2022, date last accessed).

[20] United Nations Scientific Committee on the Effects of Atomic Radiation (UNSCEAR) . Exposures of the public and workers from various sources of radiation. Report to the general assembly, Annex B. United Nations. New York: UNSCEAR, 2008.

[21] Yu-Ming L, Pei-Huo L, Ching-Jiang C et al. Measurement of terrestrial gamma radiation in Taiwan, Republic of China. Health Phys 1987;52:805–11. 3583747

[22] Chen CJ, Weng P, Chu T. Evaluation of natural radiation in houses built with black schist. Health Phys 1993;64:74–8. 8416219 10.1097/00004032-199301000-00010

[23] Nageswara MV, Bhati SS, Rama SP et al. Natural radioactivity in soil and radiation levels of Rajasthan. Radiat Protect Dosim 1996;63:207–16.

[24] Ahmad N, Matiullah KA. Indoor radon levels and natural radioactivity in Jordanian soil. Radiat Protect Dosim 1997;71:231–3.

[25] Karahan G, Bayulken A. Assessment of gamma dose rates around Istanbul (Turkey). J Environ Radioact 2000;47:213–21.

[26] Al-Jundi J, Al-Bataina BA, Abu-Rukah Y et al. Natural radioactivity concentration in soil samples along the Amman Aqaba Highway Jordan. Radiat Meas 2003;36:555–60.

[27] Egunyinka OA, Olowookere CJ, Jibiri NN et al. An evaluation of ^238^U, ^40^K, and ^232^Th concentrations in the topsoil of the University of Ibadan (UI). Southwestern Nigeria Pac J Sci Technol 2009;10:742–52.

[28] Fajemiroye JA, Adejumobi CA, Makinde SO et al. An evaluation of the soil radioactivity in the topsoil of the Polytechnic, Ibadan, southwest Nigeria. Int J Dev Sustain 2015;4:505–12.

[29] World Health Organisation (WHO) . Guidelines for Drinking Water Quality, 3rd edn ednRecommandations. Geneva, 2004, ISBN: 92-45-154638-7.

[30] Ajayi OS, Adesida G. Radioactivity in some sachet drinking water samples produced in Nigeria. Iran J Radiat Res 2009;7:151–8.

[31] Ononugbo CP, Tutumeni G. Natural radioactivity and radiation dose estimation in various water samples in Abua/Odua area, Rivers State, Nigeria. Phys sc Int J 2016;11:1–12.

[32] Adukpo OK, Faanu A, Lawluvi H et al. Distribution and assessment of radionuclides in sediments, soil and water from the lower basin of River Pra in the Central and Western Regions of Ghana. J Radioanal Nucl Chem 2015;303:1679–85.

[33] Salman AY, Kadhim SA, Alaboodi AS et al. Study the contamination of radioactivity levels of ^226^Ra, ^232^Th and ^40^K in (water) Iraq and their potential radiological risk to human population. Mater Sci Eng A 2020;928:10–26.

[34] Al-Bedri MBH . Estimation of the annual effective doses from direct ingestion of ^226^Ra and ^228^Ra in the Disi groundwater for different age groups. J Phys Conf Ser 2021;1963:1742–9.

[35] Salih NF . Measurement of natural radioactivity levels in drinking water by gamma spectrometry. Arab J Geosci 2022;15:1157–72.

[36] El-Gamal H, Sefelnasr A, Salaheldin G. Determination of natural radionuclides for water resources on the west bank of the Nile River, Assiut Governorate, Egypt. Water 2019;11:311–24.

[37] Al-Ghamdi AH . Radioactivity measurements and radiation dose assessments in ground water of Al-Baha region, Saudi Arabia. J Geosci Environ Prot 2019;7:112–9.

[38] Abdul-Rahim KS, Zainuddin Z, Idris MI et al. Determination of the radiological risk from the natural radioactivity in irrigation at selected areas of Peninsular Malaysia. Sains Malays 2020;49:1439–50.

[39] Almeida RMR, Lauria DC, Ferreira AC et al. Groundwater radon, radium and uranium concentrations in Regia ~o dos Lagos, Rio de Janeiro State, Brazil. J Environ Radioact 2004;73:323–34. 15050363 10.1016/j.jenvrad.2003.10.006

[40] Abd-Elkader MM, Shinonaga T, Sherif MM. Radiological hazard assessments of radionuclides in building materials, soils and sands from the Gaza Strip and the north of Sinai Peninsula. Sci Rep 2021;11:1–13. 34853359 10.1038/s41598-021-02559-7 PMC8636494

[41] Alzubaidi G, Hamid FBS, Rahman IA. Assessment of natural radioactivity levels and radiation hazards in agricultural and virgin soil in the State of Kedah, North of Malaysia. Sci World J 2016;2016:1–9. 10.1155/2016/6178103 PMC5124759 27965987

[42] Lu X, Pan H, Ren C et al. Natural radioactivity in reservoir sediment near an industrial park of Northwest China. J Radiol Prot 2016;36:N26–33. 27122204 10.1088/0952-4746/36/2/N26

[43] Khan HM, Ismail M, Khan K et al. Radioactivity levels and gamma-ray dose rate in soil samples from Kohistan (Pakistan) using gamma-ray spectrometry. Chin Phys Lett 2011;28:295–301.

[44] Cevik U, Damla N, Kobya AI et al. Assessment of natural radioactivity of sand used in Turkey. J Radiol Prot 2009;29:61–74. 19225183 10.1088/0952-4746/29/1/004

[45] Ramola RC, Gusain GS, Badoni M et al. ^226^Ra, ^232^Th and ^40^K contents in soil samples from Garhwal Himalaya, India, and its radiological implications. J Radiol Prot 2008;28:379–85. 18714132 10.1088/0952-4746/28/3/008

[46] Tsai T, Lin C, Wang T et al. Radioactivity concentrations and dose assessment for soil samples around nuclear power plant IV in Taiwan. J Radiol Prot 2008;28:347–60. 18714131 10.1088/0952-4746/28/3/005

